# Anna Karenina as a promoter of microbial diversity in the cosmopolitan agricultural pest *Zeugodacus cucurbitae* (Diptera, Tephritidae)

**DOI:** 10.1371/journal.pone.0300875

**Published:** 2024-04-03

**Authors:** Nele Mullens, Wouter Hendrycks, Jackline Bakengesa, Sija Kabota, Jenipher Tairo, Hannes Svardal, Ramadhani Majubwa, Maulid Mwatawala, Marc De Meyer, Massimiliano Virgilio

**Affiliations:** 1 Royal Museum for Central Africa, Biology Department, Tervuren, Belgium; 2 University of Antwerp, Department of Biology, Antwerp, Belgium; 3 Department of Crop Science and Horticulture, Sokoine University of Agriculture, Morogoro, Tanzania; 4 Department of Biology, University of Dodoma (UDOM), Dodoma, Tanzania; 5 National Sugar Institute, Academic, Research and Consultancy Section, Morogoro, Tanzania; 6 Naturalis Biodiversity Center, Leiden, Netherlands; DIU: Dhaka International University, BANGLADESH

## Abstract

Gut microbial communities are critical in determining the evolutive success of fruit fly phytophagous pests (Diptera, Tephritidae), facilitating their adaptation to suboptimal environmental conditions and to plant allelochemical defences. An important source of variation for the microbial diversity of fruit flies is represented by the crop on which larvae are feeding. However, a “crop effect” is not always the main driver of microbial patterns, and it is often observed in combination with other and less obvious processes. In this work, we aim at verifying if environmental stress and, by extension, changing environmental conditions, can promote microbial diversity in *Zeugodacus cucurbitae* (Coquillett), a cosmopolitan pest of cucurbit crops. With this objective, 16S rRNA metabarcoding was used to test differences in the microbial profiles of wild fly populations in a large experimental setup in Eastern Central Tanzania. The analysis of 2,973 unique ASV, which were assigned to 22 bacterial phyla, 221 families and 590 putative genera, show that microbial *α* diversity (as estimated by Abundance Coverage Estimator, Faith’s Phylogenetic Diversity, Shannon-Weiner and the Inverse Simpson indexes) as well as β microbial diversity (as estimated by Compositional Data analysis of ASVs and of aggregated genera) significantly change as the species gets closer to its altitudinal limits, in farms where pesticides and agrochemicals are used. Most importantly, the multivariate dispersion of microbial patterns is significantly higher in these stressful environmental conditions thus indicating that Anna Karenina effects contribute to the microbial diversity of *Z*. *cucurbitae*. The crop effect was comparably weaker and detected as non-consistent changes across the experimental sites. We speculate that the impressive adaptive potential of polyphagous fruit flies is, at least in part, related to the Anna Karenina principle, which promotes stochastic changes in the microbial diversity of fly populations exposed to suboptimal environmental conditions.

## Introduction

“True” fruit flies (Diptera, Tephritidae) include agricultural pests whose larvae attack a wide variety of crops and threaten food security at local, national and international levels [1–3]. As widely described in phytophagous insects [4–9], also in fruit flies, gut microbial communities play a pivotal role in determining insect feeding preferences. The olive fly *Bactrocera oleae* (Rossi 1790) is a classical textbook example of insect adaptation to plant allelochemicals mediated by an obligate gut symbiont [10–12]. Other than facilitating adaptation to plant allelochemical defences, microbes also contribute to fruit fly fitness traits including longevity [13], nutritional status, reproductive success [14], sexual performance [15–17], developmental rates, reproductive maturation [18], offspring development, body mass and fecundity [19]. The gut microbiome also directly or indirectly affects the fruit fly behaviour as it has been described for oviposition site selection [20] and foraging patterns [21, 22]. Last but not least, and of major importance for the containment strategies of pest species, gut microbial communities also affect insecticide resistance in fruit flies of agricultural importance [23–25].

The microbiome of tephritid fruit flies is known to be highly heterogeneous, both across and within species, with the microbial patterns of laboratory populations often deviating from those of their wild conspecifics [26, 27]. Under field conditions, an important source of variation for the insect microbial communities should be represented by the crop on which the insect larvae are feeding. However, in tephritid agricultural pests, a crop effect is not always detectable as the dominant driver of microbial diversity. In fact, other and less obvious processes [28] and the effects of high spatial heterogeneity [29, 30] interact as drivers of microbial diversity and contribute to the variability of patterns observed. The recent work of Jose et al. [28] elegantly demonstrates how crop-induced adaptation and lineage-dependent maternal effects are two interacting drivers of microbial diversity in a cosmopolitan polyphagous fruit fly. The Authors show how microbial diversity in *Ceratitis capitata* (Wiedemann) expands and contracts cyclically through the insect life stages. Bacterial *α* diversity increases in larvae due to the expansion of rare taxa, while decreases in adults, where the microbial patterns of the maternal stages “reset” to a more uniform structure across generations. Also the microbial patterns described by Jose et al. [28] are very heterogeneous and the Authors suggest that high microbial diversity might facilitate adaptation to the crop (i.e. the environment in which larvae develop), and might contribute to the insect’s polyphagous abilities.

In this paper, we try and expand this model [sensu 31] by verifying if other synergetic processes might promote fruit fly microbial diversity, and facilitate adaptive responses not only to crops but also, and more in general, to changing environmental conditions. In this context, the Anna Karenina principle [32] might represent an ecological/evolutionary process contributing to the high heterogeneity of microbial communities often observed in larval tephritids [28–30]. The Anna Karenina principle quotes the first lines of Tolstoy’s novel: “All happy families look alike; each unhappy family is unhappy in its own way”. This sentence refers to the stochastic changes induced in the microbial community by stressors, which are represented by diseases in humans [32] or, more in general by environmental stressors in wild animals [33, 34] and plants [35]. Despite occurring in a wide variety of biological systems, Anna Karenina effects (AKEs) are easily missed by the most common workflows implemented in the analysis of microbial communities and allegedly underreported in the scientific literature [32]. In fact, the analytical framework to verify the occurrence of stochastic variation promoted by AKEs relies on dedicated statistical pipelines to detect changes in the multivariate dispersion of microbial patterns [34]. To maximise chances of detecting subtle AKEs promoted by environmental stressors, and evaluate their synergetic relationships with the crop-effect we tried to minimize the effects of spatial variability [30] by focusing on a relatively small study area in Eastern Central Tanzania. For the same reasons, we targeted a model species, *Zeugodacus cucurbitae* (Coquillett) (Diptera, Tephritidae), for which exhaustive background information on distribution and life history traits is available [36–39].

*Z*. *cucurbitae* is a worldwide distributed cucurbit pest, for which incipient speciation is suspected. This species which was formerly recognised as an oligophagous fruit fly (i.e. with larvae only feeding on Cucurbitaceae), has been recorded on a more and more extended range of host plant families, including Anacardiaceae, Annonaceae, Caricaceae, Oxalidaceae, Passifloraceae, Rutaceae and Solanaceae [reviewed in 40]. The distribution, seasonal dynamics and crop preferences of *Z*. *cucurbitae* in the study area are well known as this species has been monitored in the framework of long-standing collaborative research between the Sokoine University of Agriculture and the Royal Museum for Central Africa [41–44]. The available data show a decreasing trend for crop infestations of *Z*. *cucurbitae* at higher altitudes [45], as the species gets closer to its altitudinal and thermal tolerance limits [40, 43, 46]. In the study area, and following the relevant contribution of NGOs (see Acknowledgements), cost-effective agroecological practices are being adopted by an increasing number of smallholders [47]. In the framework of ongoing projects (see Acknowledgments), and relying on the support provided by local farmers, we are comparing the differential impact of agroecological and conventional agriculture on insect biodiversity (*sensu lato*). Concerning the more specific hypotheses tested in this paper, we assume that, from an insect perspective, conventional crop management represents a more stressful environment compared to agroecological farming. This assumption is supported by the fact that (a) transient or subliminal exposure to chemical pesticides negatively affects the metabolic responses of insects which survive pesticide exposure [48–51] and (b) the use of mineral fertilizers and agrochemicals in conventional agriculture changes the soil microbial communities and, directly and indirectly, has an impact on insect microbial symbionts [52]. Conversely, we assume that agroecological farming provides comparably lower levels of environmental stress to insects due to the environmentally sustainable approach to crop protection [53]. For these reasons, we predict that conventional crop management promotes AKEs impacting the microbial patterns of *Z*. *cucurbitae*. Other than verifying if the Anna Karenina principle also applies to *Z*. *cucurbitae* we speculate about the evolutionary implications of stochastic and stress-induced changes to the symbiont microbial diversity of Tephritidae and how these changes might affect key life history traits in insects of agricultural importance.

## Materials and methods

### Field experimental setup

**Fig 1:**
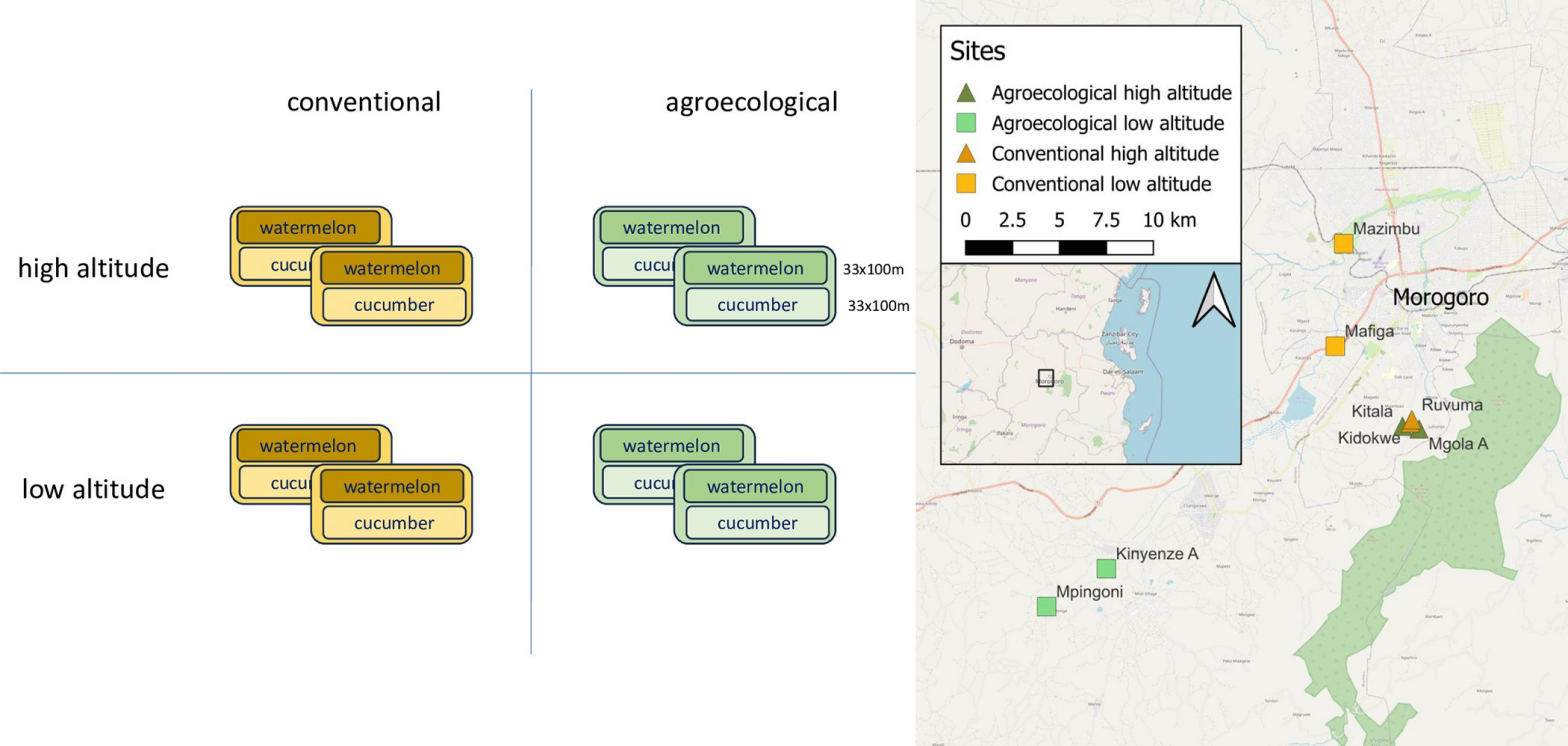
Experimental setup and map of sites (see Acknowledgments for map copyright notice).

Third instar larvae of *Z*. *cucurbitae* were collected at eight experimental sites of approximately one hectare in the Morogoro area, Eastern Central Tanzania (Fig 1, geographical coordinates provided in S1 File). Four sites were located at higher altitudes in the Uluguru mountains (~1000m elevation), other four at lower altitudes (~500m elevation) on the plains at the base of the mountains. In each site, watermelon (*Citrullus lanatus*) and cucumber (*Cucumis sativus*) were cultivated in two separated but contiguous 33x100m plots (0.33 hectares). At each altitude, agroecological management of cucurbit crops was implemented in two sites, while in the other two, conventional methods for pest control were used. Agroecological management included manual weeding, mulching, composting, no chemical control, and intercropping, while pesticides and fungicides were used for conventional crop management. The detailed protocols used in the experimental treatments are provided as Supplementary Information (S2 File). The experimental setup resulted in a balanced multifactorial design (Fig 1) including two crops (cucumber, watermelon), two altitudes (low, high), 8 sites (S1 File) and 4 replicated larval microbiomes for each combination of these factors (see below). The Sokoine University of Agriculture (SUA) approved and regulated the field site access in collaboration with the local authorities. As the Nagoya Protocol on Access and Benefit-sharing (ABS) is *de facto* not implemented in Tanzania, the intellectual and physical property of samples collected in this study is regulated by Mutually Agreed Terms (MATs) on the use of genetic resources established between SUA and RMCA. This document, which is inspired and fully adheres to the principles of the Nagoya protocol, is provided as supplementary S3 File.

### Identification and microbial profiling of wild larvae

Before the beginning of the short rainy season [44], between 2–9 November 2021, 5 infested cucumbers and 5 infested watermelons were collected in each experimental site. Third instar larvae (0 to 20 per fruit) were collected after dissecting the fruits at the Horticultural Unit of SUA, rinsed in phosphate-buffered saline solution (PBS) and preserved in individual tubes at -20°C in 98% ethanol (EtOH). Since the morphological identification (ID) of larvae is highly problematic [54], DNA barcoding following the methods detailed in Virgilio et al. [55] was used for larval ID. Full body DNA extraction [see 56] was implemented on all collected larvae using the DNeasy Blood and Tissue kit (Qiagen Inc., Hilden, Germany). Of all larvae identified, 4 larvae of *Z*. *cucurbitae* were subsampled in each site, for each crop by randomly selecting four specimens from each of the available batches of *Z*. *cucurbitae*). Their microbial patterns were characterised via DNA metabarcoding of the V3 and V4 regions of 16S rRNA as described in Hendrycks et al. [29]. After quality checking of the raw data using Fastqc [57], the DADA2 pipeline [58] was used for read filtering, trimming, demultiplexing and recovering Amplicon Sequence Variants (ASVs). Following Bell et al. [59], a negative control was included in the analysis and used to correct for contamination bias via microDecon [60]. We used the Silva v132 reference database [61] for the taxonomic assignment of the ASVs to phylum, family and genus level and to discard non-bacterial sequences such as mitochondria and chloroplast from the dataset.

### Data analysis and hypothesis testing

We relied on a common hypothesis testing framework for both a and β microbial diversity. To help ensure robust biological interpretations we adopted a consensus approach based on multiple methods to infer differential bacterial abundances [62]. Microbial α diversity was calculated after standardising ASVs counts into relative frequencies per sample and estimated via the Abundance Coverage Estimator (ACE), the Faith’s Phylogenetic Diversity index (PD) the Shannon-Weiner index (H) and the Inverse Simpson index (IS). The phylogenetic tree on which PD was based, was aligned using DECIPHER [63] and constructed with RAxML BlackBox [64], implementing RAxML-HPC v.8 with 400 bootstraps on the CIPRES Science Gateway v.3.3 portal (https://www.phylo.org) [65]. Microbial β diversity was estimated considering the differential abundances of (a) 4,548 filtered ASVs and (b) 430 aggregated bacterial genera (S1 Table) identified via DADA2. All reads which could not be assigned to genus level after cross-matching with the Silva reference database were aggregated into distinct groups, each including all NAs belonging to the same family. Each of these groups was considered as a proxy for an unidentified genus. Inference on β diversity mainly relied on compositional data analysis (CoDa) based on centered log-ratio (CLR) transformed data [62]. The robustness of patterns observed through CoDa was also verified by repeating the analyses on ASVs frequencies [62, 66, 67]. In this context, different transformations of ASVs frequencies were implemented during Permutational Multivariate Analysis of Variance (PERMANOVA, see below) to modulate the weight of dominant taxa and to better detect possible changes in the abundance of rare taxa [68]. Here we report results for untransformed data, fourth-root, log(X+1) and presence/absence transformed data (in order of increasing weight given to the less abundant taxa). Differences in α diversity between management practice (Ma: conventional vs agroecological), altitude (Al: high vs low), crop (Cr: watermelon vs cucumber), and site (Si, see S1 File) were tested by Analysis of Variance (ANOVA) as implemented by GAD [69], with Al, Cr and Ma as fixed, orthogonal factors and Si as a random factor nested in (Al x Ma). Homoscedasticy was preliminarily verified via Cochran’s C test, the data transformed when required (Underwood 1997) the Student- Newman-Keuls (SNK) test was used for *a posteriori* comparisons of means (Sokal & Rohlf, 1995). Location and dispersion effects on β diversity [see 34] were tested via PERMANOVA and Permutational Multivariate Analysis of Dispersion [70] as implemented in Primer-e 7.0.21 [71]. PERMANOVA and PERMDISP on CLR transformed data were based on Euclidean Distances (as allowing negative values), while PERMANOVA on ASVs frequencies on Bray-Curtis distances [68]. PERMANOVA was based on 999,999 permutations of residuals under a reduced model and on the same 4-factor experimental design (Cr, Al, Ma, Si) considered for α diversity. *A posteriori* pairwise comparisons of significant interactions of factors were implemented via permutational t-statistics [70].

As the analyses of both α and *β* diversity indicated a significant interaction of Al and Ma (see Results), and as PERMDISP only allows for single-factor tests, we separately verified differences in patterns of multivariate dispersions at high and low altitudes. The probability values of repeated tests were corrected using the False Discovery Rate (FDR) procedure [72]. Patterns of β diversity were visualised using unconstrained ordination [Principal Coordinates Analysis, PCO, 73, 74]. ALDEx2 [75] was used to test differential abundances of bacterial genera between management practices at high altitude and allowed the detection of bacterial genera which significantly contributed to the above-mentioned differences in β diversity (see Results). The ALDEx2 analyses, relied on CRL transformed data, so to maintain the CoDA approach already implemented in PERMANOVA and PERMDISP. As recommended by Gloor [76], taxa which showed an effect size difference between 1 and −1 were filtered out to reduce biases due to false positives. Differential abundances of bacterial genera were tested by the Welch t-test (as more restrictive than the Wilcoxon rank‐sum test also available in ALDEx2) followed by FDR correction [72]. A graphical overview of the analytical pipeline can be found in S1 Graphical abstract. The raw sequencing data have been deposited in the European Nucleotide Archive (ENA) with accession number PRJEB70707. The complete bioinformatic pipeline (also including the scripts used in DADA2, microDecon and ALDEx2) can be downloaded from https://zenodo.org/doi/10.5281/zenodo.10520034.

## Results

**Fig 2:**
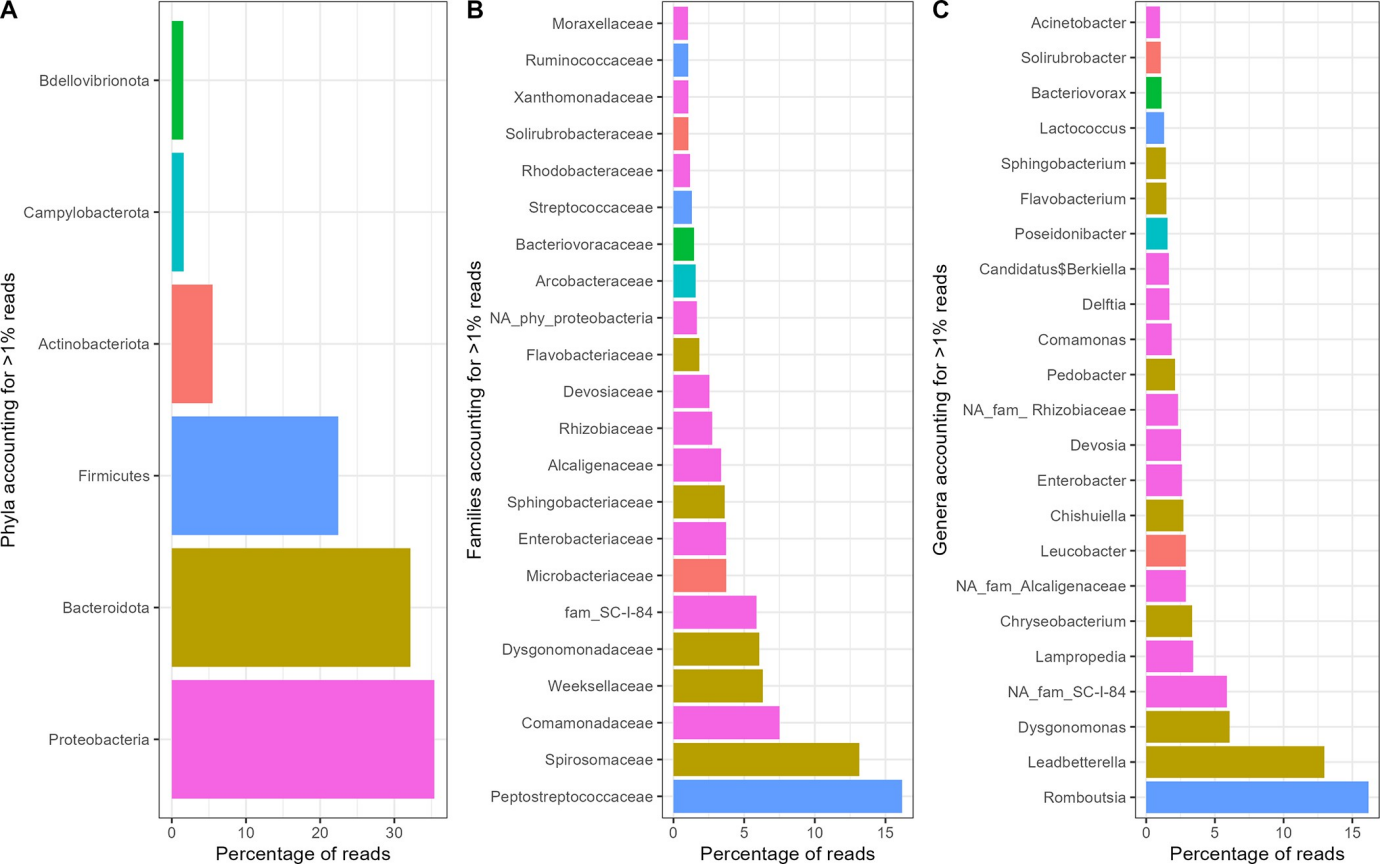
Most abundant (i.e. > 1% of reads) bacterial (A) phyla, (B) families and (C) genera across the experimental treatments. Colours in B and C refer to phylum classification.

The MiSeq run produced more than 1.0×10^7^ raw reads (average number reads / sample = 166,311, SD = 23,694) which after filtering were reduced to 3.0×10^6^. The resulting 2,973 unique ASV were assigned to 22 phyla, 221 families and 590 putative genera. These latter included 113 unidentified genera represented by 1–94 ASVs from the same family and including 15.45% of filtered reads (see methods). The five most abundant (here defined as representing > 5% of reads) phyla were Proteobacteria (35,39% of reads, including 63 families), Bacteroidota (32,16% of reads, 28 families), Firmicutes (22.43% of reads, 37 families) and Actinobacteriota (5,50% of reads, 38 families). The most abundant families included Peptostreptococcaceae (phylum Firmicutes, 2 genera, 16,17% of reads), Spirosomaceae (Bacteroidota, 10 genera, 13,15% of reads), Comamonadaceae (Proteobacteria, 28 genera, 7,50% of reads) and Weeksellaceae (Bacteroidota, 12 genera, 6,32% of reads). The most abundant genera included *Romboutsia* (family Peptostreptococcaceae, 16,17% of reads), *Leadbetterella* (Spirosomaceae, 12,96% of reads), *Dysgonomonas* (Dysgonomonadaceae, 6,07% of reads) and an unidentified genus representing 5,87% of reads from taxon SC-I-84 (NCBI:txid102458). An extended list of abundant phyla, families, and genera (> 1% of reads) is represented in Fig 2. A complete overview of the abundances of the aggregated genera is provided in S1 Table.

**Fig 3:**
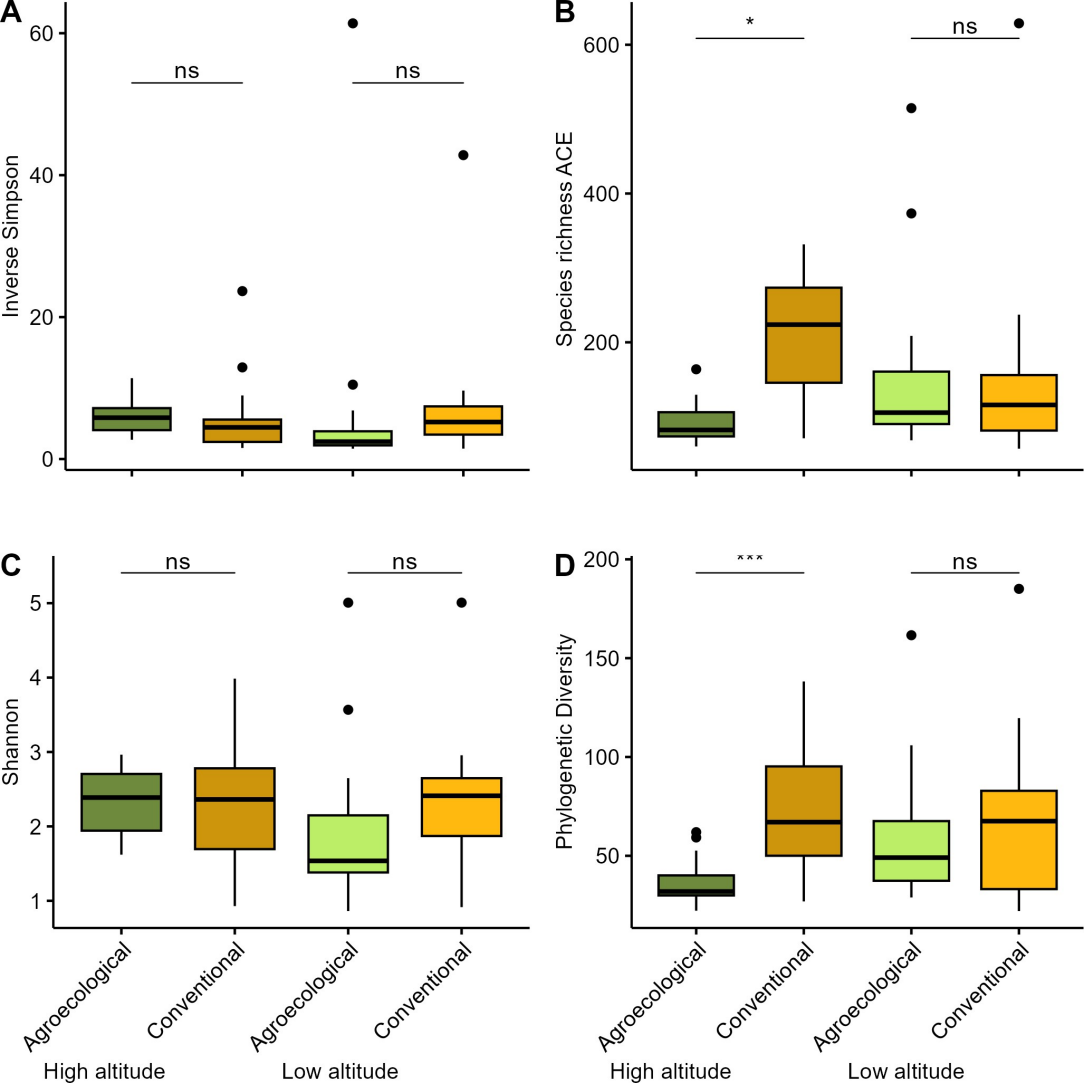
Differences in microbial α diversity between agroecological and conventional farming at low and high altitude. (A) Inverse Simpson index (IS), (B) Abundance Coverage Estimator (ACE), (C) Shannon-Weiner index (H) and(D) Faith’s Phylogenetic Diversity (PD). Significant differences as detected by ANOVA are indicated.

ANOVA (Table 1) showed a significant interaction between Management Practice and Altitude for both ACE (F_1, 4_ = 12.212, P = 0,025) and PD (F_1, 4_ = 22.237, P = 0.009). The *posthoc* comparisons (Table 1) revealed that, at high altitude, species and phylogenetic richness were higher in microbial communities from conventional compared to agroecological management practices, while no significant differences were observed at low altitude (Fig 3B and 3D). Additionally, a significant interaction between Crop and Site was observed for both ACE (F_4, 48_ = 5.312, P = 0.001) and PD (F_4, 48_ = 5.817, P = 0.001). Neither H nor IS showed significant differences across all the factors tested (Fig 3A and 3C).

**Table 1:** Hypothesis testing framework and consensus approach (see methods) to verify differences in α and β microbial diversity (detailed results in S2 Table).

Location effects	α diversity–ANOVA (ASVs frequencies)				β diversity–PERMANOVA (CLR-CoDa)		β diversity–PERMANOVA (ASVs frequencies)			
	ACE	PD	H	IS	CLR ASVs	CLR aggregated genera	untransformed	4rt root	– log(X+1)	presence/absence
Management Practice: Ma	*	**								
Altitude: Al										
Crop: Cr										*
Ma x Al	*	**			*	*	*	*	*	*
Al x Cr										
Ma x Cr										
Site: Si (Ma x Al)					***	***	***	***	***	***
Al x Ma x Cr										
Cr x Si (Ma x Al)	**	***			***	***	***	***	***	***
*posthoc* test Ma x Al										
high altitude	conv. > agroec.	Conv. > agroec.			Conv. ≠ agroec.	Conv. ≠ agroec.				
Low altitude	conv. = agroec.	Conv. = agroec.			Conv. = agroec.	Conv. = agroec.				
**Dispersion effects (Anna Karenina)**					β diversity–PERMDISP (CLR-CoDa)					
					CLR ASVs	CLR aggregated genera				
Management Practice, High					conv. > agroec.	Conv. > agroec.				
Management Practice, Low					conv. = agroec.	Conv. = agroec.				

ANOVAs (on ASVs frequencies) and PERMANOVAs (on either ASVs frequencies or centered log-ratio transformed, compositional data, CLR-CoDa) were used to test univariate and multivariate location effects of management practice (Ma: conventional vs agroecological), altitude (Al: high altitude vs low altitude), crop (Cr: watermelon vs cucumber), and site (list and coordinates in S1 File). PERMDISP, based on CLR-CoDa, was used to test dispersion effects on β diversity (Anna Karenina principle) promoted by different management practices either at high or low altitudes. FDR: p value corrected via False Discovery Rate. Tests on α microbial diversity considered Abundance Coverage Estimator (ACE), Faith’s Phylogenetic Diversity (PD), Shannon-Weiner (H) and the Inverse Simpson indexes (IS). The results of the *post hoc* tests implemented for Ma x Al are indicated.

*: P = <0.05

**: P = <0.001

***: P = <0.0001.

**Fig 4:**
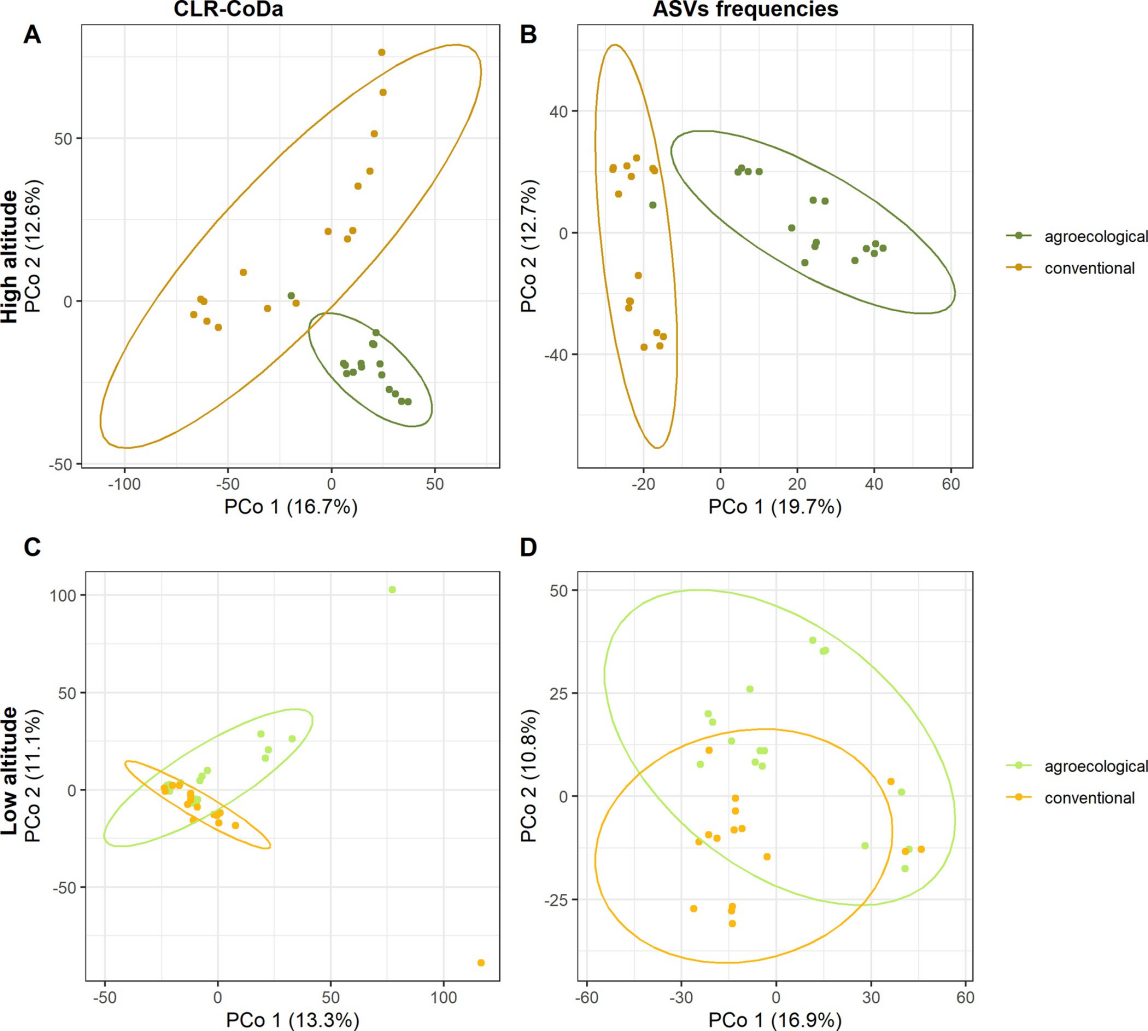
Principal Coordinates Analysis (PCoA) of the microbial communities observed in agroecological and conventional farming at low and high altitude. **Results are based on either ASVs frequencies or centered log-ratio transformed, compositional data (CLR-CoDa).** For the different groups, 95% confidence ellipses are indicated.

PERMANOVA (Table 1, S1 Table) showed a significant interaction between Management Practice and Altitude in the compositional data analysis of both ASVs (pseudo-F_1, 4_ = 1.787, P = 0.026) and of bacterial genera (pseudo-F_1, 4_ = 1.984, P = 0.044). This interaction represented 10,46% of the estimated components of variation for ASVs and 12,85% for bacterial genera. For both ASVs and bacterial genera, the *posthoc* comparisons detected significant differences only between conventional and agroecological management at high altitude, while no significant differences were observed at low altitude (Fig 4). Also Site and the interaction between Crop and Site were detected as highly significant effects, contributing to 13,45% and 18,74% to the estimated components of variation in the analysis of ASVs and to 15,17% and 22,56% in the analysis of bacterial genera. We could observe highly consistent patterns (with the interaction between Management Practice and Altitude, Site and the interaction between Crop and Site detected as highly significant effects) when considering ASVs frequencies and frequencies of bacterial genera across all transformations of data implemented (untransformed, fourth-root, log(X+1), presence/absence) (**S2** Table).

At high altitude, PERMDISP showed significantly higher multivariate dispersion for conventional (average Euclidean distance from centroid = 81.12, SE = 3.304) compared to agroecological management (average Euclidean distance = 60.49, SE = 2.632). Conversely, at low altitude, the microbial communities from conventional (average Euclidean distance = 76.03, SE = 5.68) or agroecological management (average Euclidean distance = 71.32, SE = 5.90) did not show significant variation (Fig 4).

**Fig 5:**
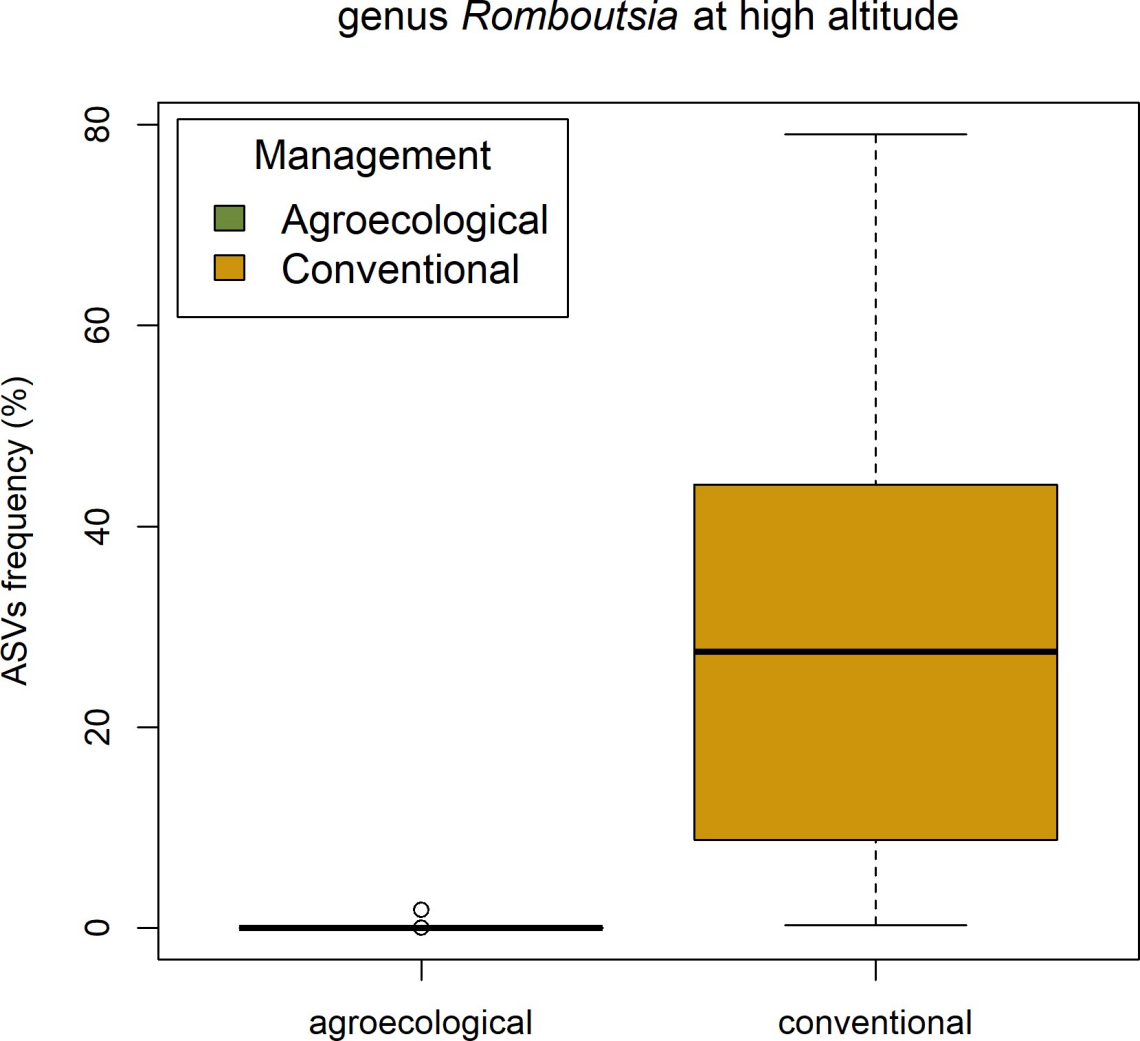
Abundance of *Romboutsia* in conventional and agroecological farming at high altitude.

These bacterial patterns at high altitude were further investigated with ALDEx2 (S3 Table) which showed that five genera have significant differential abundances between conventional and agroecological management. Among these, is *Romboutsia*, the most abundant bacterial genus detected in our study system (Fig 5).

**Fig 6:**
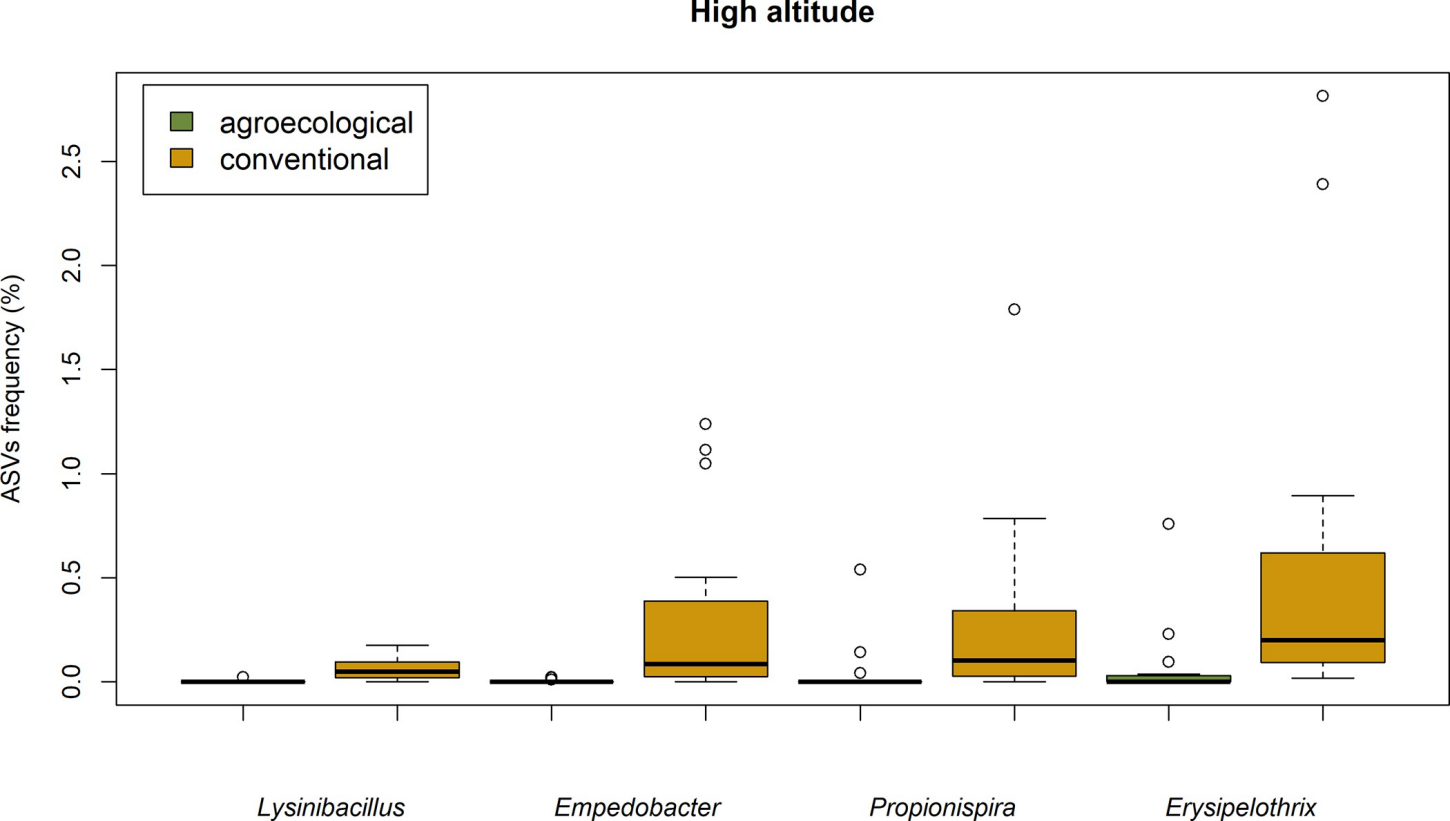
Abundance of *Lysinibacillus*, *Empedobacter*, *Propionispira* and *Erysipelothrix* in conventional and agroecological farming at high altitude.

Significantly different distributions were also detected for other four genera with abundances < 1%. These included *Lysinibacillus* (ASVs frequency = 0.056%), *Empedobacter* (0.077%), *Propionispira* (0.16%) and *Erysipelothrix* (0.46%) (Fig 6).

## Discussion

The data presented in this study further suggest how the concept of “core microbiome” only loosely applies to tephritids [30]. As observed for other fruit fly species, the microbial community patterns of *Z*. *cucurbitae* reported in the literature are quantitatively and qualitatively heterogeneous [27, 29, 30, 77–84]. Asimakis et al. [85] reported how Enterobacteriaceae, Dysgomonadaceae and Orbaceae were dominant families in field populations of *Z*. *cucurbitae* from Bangladesh, with genera *Dysgonomonas*, *Orbus* and *Citrobacter* occurring in relatively high abundances across populations. De Cock et al. [30] suggested that the genus *Ochrobactrum* might be a core representative of the microbiome of *Z*. *cucurbitae*. Hendrycks et al. [29], used a more rigorous statistical framework [86] on larvae collected from the Morogoro area, from crops including those targeted by this study (watermelon and cucumber); they identified *Enterobacter*, *Klebsiella*, *and Citrobacter* as core genera for *Z*. *cucurbitae*. Five of these genera were also observed in this study, yet only *Dysgonomonas* and *Enterobacter* in relatively high frequencies, while *Citrobacter*, *Ochrobactrum* and *Klebsiella* occurred in low frequencies (see S1 Table). Conversely, two very abundant bacterial genera in our experimental setup, *Romboutsia* and *Leadbetterella*, which contributed to about 29% of all ASVs, were not mentioned as abundant taxa by previous research on larval *Z*. *cucurbitae*. Similarly, Enterobacteriaceae, which are described as a very abundant family in other studies and contributing up to 90% of reads [30, 78, 87, 88] occurred in much lower proportions (3.67% of reads).

Multiple factors contribute to the variability commonly observed in laboratory strains as well as in wild fruit fly populations [27, 89, 90]. Some of them are obvious and include biases related to heterogeneous sampling, manipulation and preservation procedures [56]. However, the high variability observed across studies describing insect microbial communities also originates from heterogeneous and non-standardised approaches to data analysis. These include combinations of (a) varying assumptions on data distributions [62], (b) differential abundance testing methods [62, 91, 92], and (c) data filtering strategies [93, 94], including the much-debated data rarefying [95]. A widely used approach in the analysis of microbial communities is to consider data from microbial analyses as compositional [67, 75, 96–98] and providing information on the abundance of bacterial taxa in relation to the other taxa occurring in the very same dataset [62, 99]. Accordingly, and to detect subtle changes in the microbial patterns of *Z*. *cucurbitae*, we focused our hypothesis testing framework on both a *self-contained* study system (a large experimental setup in Central Eastern Tanzania) and on an analytical framework largely based on centered log-ratio transformed, compositional data. The robustness of the patterns observed was also supported by more conventional statistical procedures including the analysis of bacterial frequencies [as previously done in 29, 30]. For the reasons stressed in [62, 91, 92], we also limited to the minimum inference based on cross-comparisons with studies dealing with fruit fly microbial abundances.

The data collected through the consensus approach [sensu 62] adopted in this study all show that the microbial communities of *Z*. *cucurbitae* are affected by the combined effects of management practices and altitude. These two drivers of microbial diversity have a stronger, synergetic effect in conventional farming at high altitude. The effects promoted by management practices across altitudes, crops, experimental sites (i.e. as a stand-alone factor), are very clear on β diversity but comparably subtler on α diversity. In fact, higher diversity in conventional management practices could only be detected by two of the α diversity estimators (ACE, PD). Regardless of that, and as observed for β diversity, these metrics confirmed the occurrence of higher diversity in conventional farming at high altitude. The crop effect on the microbial communities of *Z*. *cucurbitae* (as measured in terms of both α and β diversity) was also relatively weak and only detectable as not consistent changes across the experimental sites (as showed by the significant interaction of Crop and Site). These results further confirm the patterns already observed in other studies targeting wild populations of *Z*. *cucurbitae*, which showed strong random variability at regional [30] or local spatial scales [29].

Interestingly, the most abundant bacterial genus in our study system, *Romboutsia*, occurs in higher abundance in larvae from conventional farming. In vertebrates, *Romboutsia* (among other bacteria) has been described as a key genus mediating physiological responses to agrochemicals. Liu et al. [100] observed changes in the abundance of *Romboutsia* in mice exposed to fungicides and highlighted how this genus is involved in metabolic pathways such as the production of amino acids, free fatty acids and their methyl esters, phospholipids, nucleotides, carbohydrates and hormones. Similarly, Yang et al. [101] described relationships between the abundance of *Romboutsia* and exposure to Deltamethrin, a commonly used pesticide in the Morogoro area. These results suggest that *Romboutsia*, and possibly also other genera occurring with differential frequencies in conventional and agroecological farming at high altitude (*Lysinibacillus*, *Empedobacter*, *Propionispira*, *Erysipelothrix*) might be implicated in the responses of *Z*. *cucurbitae* to stressors. But of course, our descriptive analysis does not allow further speculation, and the possible role of these microbial groups in affecting the metabolic pathways of *Z*. *cucurbitae* will require targeted experimental support.

This study also highlights how the patterns of microbial β diversity of *Z*. *cucurbitae* are subjected to changes in multivariate dispersion. Also in this case, these changes are only detectable at high altitude where comparably higher dispersion is observed in conventional rather than in agroecological farming. The biological interpretation of these patterns indicates that the microbial communities of *Z*. *cucurbitae* follow the Anna Karenina principle [34]. In this context, AKEs would promote the microbial diversity of populations of *Z*. *cucurbitae* which are exposed to more stressful environmental conditions. In this specific case, closer to the altitudinal limits of *Z*. *cucurbitae* in the Morogoro area [40, 43, 45, 46] and in farms where pesticides and agrochemicals are used [48–51]. As reported for laboratory populations of the closely related genus *Bactrocera*, insecticide toxicity is significantly affected by temperature [102–104]. Accordingly, pesticides applied at higher altitudes in the Morogoro area might be more effective as acting on larvae exposed to the suboptimal environmental conditions promoted by lower temperatures. We suggest that the significantly higher α diversity observed in larvae from these farms also reflects the stochastic changes promoted by AKEs in stressed larvae. These results might also be in line with those of Jose et al. [28] who reported higher α diversity in medfly larvae feeding on different fruits and lower diversity in adult mothers (in which they observed a strong bias towards high abundance of few bacterial species). We speculate that the effects related to fruit host in Jose et al. [28] might also have been affected by AKEs, as larvae feeding on heterogeneous crops are allegedly subjected to heterogeneous levels of environmental stress.

## Conclusions

We speculate that AKEs might promote adaptation in Tephritidae at micro- and macro-evolutionary scales. In this context, the stochastic “boost” of microbial diversity promoted by the Anna Karenina principle would be beneficial under changing environmental conditions as it would maximise chances that suitable bacteria, occurring within the microbial pool, could contribute to the insect responses to stress. If the generality of patterns observed in *Z*. *cucurbitae* would also be confirmed in other fruit flies, then, AKEs might explain at least part of the impressive adaptive potential observed in Tephritidae, a family of notorious agricultural pests for which rapid adaptation to unsuitable host plants [29, 40], sudden range expansions [105, 106], and host race formation and speciation [107–109] have been described.

## Supporting information

S1 File Geocoordinates, altitudes and crop management of sites included in the experimental setup. (DOCX)

S2 File Experimental protocols implemented for the agroecological and conventional management of cucurbit crops. (DOCX)

S3 File Mutually Agreed Terms (MATs) on the use of genetic resources established between SUA and RMCA. (PDF)

S1 Graphical abstract Graphical overview of the analytical pipeline. (PNG)

S1 Table Frequencies of aggregated genera (% of ASVs) in order of abundance. Light green: very abundant taxa (above 5%), light blue: abundant taxa (above 1%), grey: rare taxa (below 1%).
(XLSX)

S2 Table Hypothesis testing framework and consensus approach to verify differences in α and β microbial diversity. ANOVAs (on ASVs frequencies) and PERMANOVAs (on either ASVs frequencies or centered log-ratio transformed, compositional data, CLR-CoDa) were used to test univariate and multivariate location effects of management practice (Ma: conventional vs agroecological), altitude (Al: high vs low), crop (Cr: watermelon vs cucumber), and site (list and coordinates in S1 File). PERMDISP, based on CLR-CoDa, was used to test dispersion effects on β diversity (Anna Karenina principle) promoted by different management practices either at high or low altitudes. FDR: p value corrected via False Discovery Rate. Tests on α microbial diversity considered Abundance Coverage Estimator (ACE), Faith’s Phylogenetic Diversity (PD), Shannon-Weiner (H) and the Inverse Simpson indexes (IS). The results of the *posthoc* tests implemented for Ma x Al are indicated. *: P = <0.05; **: P = <0.001; ***: P = <0.0001. For detailed results see sheets "ANOVA", PERMANOVA", "PERMDISP".
(XLSX)

S3 Table ALDEx2 results detecting which bacterial genera significantly contributed to the differences observed in β diversity between management practices at high altitude. Taxa with effect size difference between 1 and −1 were filtered out to reduce biases due to false positives. Differential abundances of bacterial genera were tested by the Welch t test (as more restrictive than the Wilcoxon rank‐sum test also available in ALDEx2) followed by FDR correction (Benjamini & Hochberg, 1995).
(XLSX)
