# Supplementary material for: Anna Karenina as a promoter of microbial diversity in the cosmopolitan agricultural pest Zeugodacus cucurbitae (Diptera, Tephritidae)
Source: PLoS One. 2024 Apr 3;19(4):e0300875. doi: 10.1371/journal.pone.0300875 (PMC10990204; doi:10.1371/journal.pone.0300875)
Supplement: S1 File — (DOCX) [file pone.0300875.s001.docx]

| **site name** | **altitude m** | **management of cucurbit crops** | **geocoordinates** |
| --- | --- | --- | --- |
| Kinyenze A | 555 | agroecological | 6°56'45.7"S, 37°31'16.2"E |
| Mpingoni | 597 | agroecological | 6°57'51.0"S, 37°29'32.6"E |
| Mgola A | 1,069 | agroecological | 6°52'45.1"S, 37°40'18.8"E |
| Kitala | 1,029 | agroecological | 6°52'40.5"S, 37°39'50.2"E |
| Mafiga | 503 | conventional | 6°50'22.8"S, 37°37'53.5"E |
| Mazimbu | 486 | conventional | 6°47'26.2"S, 37°38'07.9"E |
| Kidokwe | 960 | conventional | 6°52'34.6"S, 37°40'03.7"E |
| Ruvuma | 995 | conventional | 6°52'29.3"S, 37°40'06.5"E |
