# Supplementary material for: Anna Karenina as a promoter of microbial diversity in the cosmopolitan agricultural pest Zeugodacus cucurbitae (Diptera, Tephritidae)
Source: PLoS One. 2024 Apr 3;19(4):e0300875. doi: 10.1371/journal.pone.0300875 (PMC10990204; doi:10.1371/journal.pone.0300875)
Supplement: S2 File — (DOCX) [file pone.0300875.s002.docx]

1. **Conventional management**

- NPK (N:P:K = 15:9:20) commercial fertilizer, 102 kg/ha 14 days after seed emergence
- NITRABOR (15.4%N+26%Ca+0.3%B), 102 kg/ha starting from the flowering stage, every two weeks
- commercial insecticide (organophosphate and pyrethroids), 2.5 L/ ha every two weeks
- commercial fungicide (Chloroforce, chlorothalonil) 2.5 L/ ha every two weeks

1. **Agroecological management (NGO “Sustainable Agroecology Tanzania)**

- organic-insecticide twice a week (700g neem leaves, 200g of chili, 100g garlic, two aloe vera leaves, 4 spoons of cooking oil). One kg mixture into10L of water for 12 hours.
- organic fungicide twice a week (1lLmilk, 100g baking powder, 10L of water)
- organic fungicide once/season (1kg of grided pawpaw leaves, 10L of water)
- organic fertilizers 250ml / week / plant (50kg fresh cow dung, 200L of water, 21 days).
- 0.5kg of cow manure per plant hole before planting
- mulching layer of 15 cm (dried straw)
- intercropping using green gram, 50cm spacing.
- organic fencing surrounding the experimental sites (pigeon peas)
