# Supplementary material for: Anna Karenina as a promoter of microbial diversity in the cosmopolitan agricultural pest Zeugodacus cucurbitae (Diptera, Tephritidae)
Source: PLoS One. 2024 Apr 3;19(4):e0300875. doi: 10.1371/journal.pone.0300875 (PMC10990204; doi:10.1371/journal.pone.0300875)
Supplement: S3 File — (PDF) [file pone.0300875.s003.pdf]

Mutually Agreed Terms on the Use of Genetic Resources collected within the framework of the ISeBAF project.

This agreement is between:

Sokoine University of Agriculture (SUA), Morogoro, Tanzania

and

Royal Museum for Central Africa (RMCA), Tervuren, Belgium

Whereas:

ISeBAF is the acronym of a collaborative project between SUA and RMCA, funded by the Belgian Sicence Policy (BELSPO) through the Belgian Research Action through Interdisciplinary Networks PHASE 2 - 2018-2023 (BRAIN-be 2.0).

ISeBAF aims at conducting research on tephritid fruit flies (Diptera: Tephritidae) and dipteran and hymenopteran pollinators (several families) related to the production of cucurbit and solanaceous crops in different parts of Tanzania.

ISeBAF will, within the framework of the project, collect Diptera and Hymenoptera specimens which will be preserved by means of different technologies. These specimens, or any parts or products forthcoming from these and containing genetic material, shall be considered within this agreement as genetic resources and is hereafter referred to as 'material'.

It is agreed that:

1. All material collected in Tanzania within the framework and duration of the ISeBAF project shall remain the property of the SUA. The material collected shall be intended for scientific research and shall have no commercial purposes.
2. All or part of the collected material, can be transferred to RMCA for accurate species identification and other research activities according to the research strategy and methodologies detailed in the Technical Specifications of contract n. B2/191/P1/ISeBAF BRAIN-be 2.0 (Annex I).
3. Transfer of materials shall be conducted upon mutual agreement of both parties and shall be within the framework of necessary scientific activities that require transfer of materials.
4. All or part of the transferred material can be stored and registered in the RMCA collections as voucher specimens for future reference as well as for possible additional use in the framework of collaborative research activities developed by both parties (SUA and RMCA).
5. All results, forthcoming from research conducted on the transferred material, shall be the property of both SUA and RMCA.
6. Non-commercial use of these results shall be the subject of specific ad hoc agreement between the SUA and RMCA researchers involved in the generation of these results.
7. Commercial use of the material transferred to RMCA, or of the results forthcoming from research conducted on the transferred material, shall not be allowed without prior consent of SUA and shall not be covered by this agreement.
8. Where applicable, SUA will ensure that the national and international regulations are followed regarding legal collecting and transfer of biological specimens.
9. All consumables and equipment purchased within the framework of this agreement and through the budget provided, can be used solely for the activities listed in Annex I. Where applicable, equipment purchased will remain property of SUA after finalization of the project.

Signed on 18/11/2021

For RMCA

Dr Massimiliano Virgilio
RMCA Promoter

For SUA

Prof. Ramadhani Majubwa
SUA Promoter
