# Supplementary figures and images for: Anna Karenina as a promoter of microbial diversity in the cosmopolitan agricultural pest Zeugodacus cucurbitae (Diptera, Tephritidae)
Source: PLoS One. 2024 Apr 3;19(4):e0300875. doi: 10.1371/journal.pone.0300875 (PMC10990204; doi:10.1371/journal.pone.0300875)

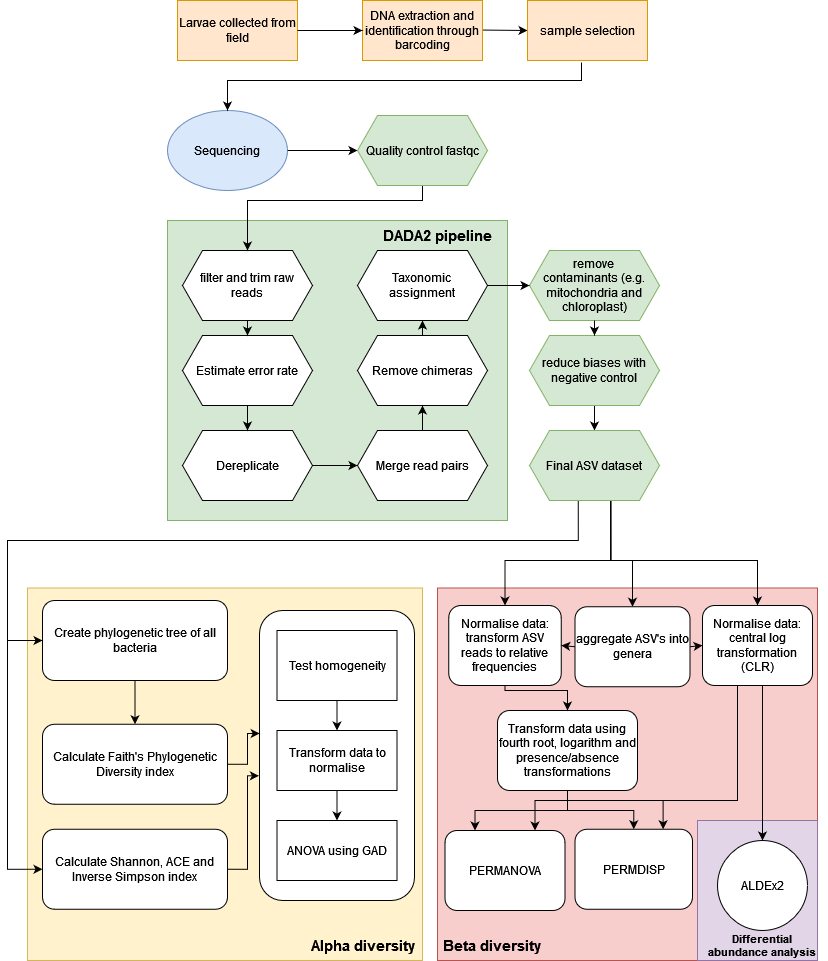

Supplement: S1 Graphical abstract — (PNG) [file pone.0300875.s004.png]
